# Factors Affecting Population Dynamics of Maternally Transmitted Endosymbionts in *Bemisia tabaci*

**DOI:** 10.1371/journal.pone.0030760

**Published:** 2012-02-23

**Authors:** Huipeng Pan, Xianchun Li, Daqing Ge, Shaoli Wang, Qingjun Wu, Wen Xie, Xiaoguo Jiao, Dong Chu, Baiming Liu, Baoyun Xu, Youjun Zhang

**Affiliations:** 1 Department of Plant Protection, Institute of Vegetables and Flowers, Chinese Academy of Agricultural Sciences, Beijing, China; 2 Department of Entomology and BIO5 Institute, University of Arizona, Tucson, Arizona, United States of America; 3 High-tech Research Center, Shandong Academy of Agricultural Sciences, Jinan, China; Ghent University, Belgium

## Abstract

While every individual of *Bemisia tabaci* (Hemiptera: Aleyrodidae) harbors the primary symbiont (P-symbiont) *Portiera*, the infection frequencies of the six secondary symbionts (S-symbionts) including *Hamiltonella*, *Arsenophonus*, *Cardinium*, *Wolbachia*, *Rickettsia* and *Fritschea* vary greatly among different populations. To characterize the factors influencing the infection dynamics of the six S-symbionts in *B. tabaci*, gene-specific PCR were conducted to screen for the presence of the P-symbiont *Portiera* and the six S-symbionts in 61 (17 B and 44 Q biotypes) field populations collected from different plant species and locations in China. All individuals of the 61 populations hosted the P-symbiont *Portiera*, but none of them harbored *Arsenophonus* and *Fritschea*. The presence and infection rates of *Hamiltonella*, *Cardinium*, *Rickettsia, Wolbachia* and their co-infections *Rickettsia + Hamiltonella (RH), Rickettsia + Cardinium (RC), Hamiltonella + Cardinium (HC)* and *Rickettsia + Hamiltonella + Cardinium (RHC)* varied significantly among the 61 field populations; and the observed variations can be explained by biotypes, sexes, host plants and geographical locations of these field populations. Taken together, at least three factors including biotype, host plant and geographical location affect the infection dynamics of S-symbionts in *B. tabaci*.

## Introduction

Bacteria commonly form intimate, symbiotic associations with insects. These bacterial endosymbionts occur in a diverse array of insect species and are usually passed from host to host by vertical transmission [1], [2]. The endosymbionts of insects are divided into two groups: primary symbionts (P-symbionts) and secondary symbionts (S-symbionts) [1]. P-symbionts often produce/provide essential nutrients for their hosts and thus form an obligatory mutualistic relationship with their hosts, i.e., they depend on each other for survival. Examples include the bacterial symbionts that make nutrients for their insect hosts, such as *Buchnera aphidicola* in aphids, *Carsonella ruddii* in psyllids, and *Tremblaya princeps* in mealybugs [1]. By contrast, S-symbionts may not be required for host survival. Nonetheless, they may play important roles in their host's nutrition [3], [4], genetic differentiation [5], adaptation to a wide range of food plants [6], defense against natural enemies [7]–[9], reproduction [10], [11], sensitivity to heat stress and other environmental factors [12].

The whitefly *Bemisia tabaci* (Gennadius) (Hemiptera: Aleyrodidae) is one of the most destructive insect pests of numerous protected and field crops worldwide. It causes serious damage in many crops by direct feeding and by vectoring 111 plant viruses [13]. *B. tabaci* has long been thought to comprise morphologically indistinguishable biotypes that often differ in host range, fecundity, insecticide resistance, and/or transmission competency for begomoviruses [14], [15]. Recent studies suggest that most of these biotypes represent genetically distinct cryptic species [16]–[19]. The B biotype of the Middle East-Minor Asia 1 and the Q biotype of Mediterranean group are among the most invasive and destructive biotypes [16], [19], [20]. *B. tabaci* was first recorded in the late 1940s in China, but the crop damages and loses caused by this insect had not been serious until the introduction of the B biotype in the 1990s [21]. In recent years, the Q biotype of *B. tabaci* has invaded China [22], and in many areas, even displaced the B biotype [23].

To date, one P-symbiont and six S-symbionts have been reported from *B. tabaci* [1]. The P-symbiont is *Portiera aleyrodidarum*, which supplements nutrients that *B. tabaci* can not obtain sufficient amounts from a restricted diet of plant phloem [1], [24]. The six S-symbionts are *Hamiltonella* [25], *Arsenophonus* [26], *Cardinium* [27], *Wolbachia* [25], *Rickettsia* [28] and *Fritschea* [29]. These S-symbionts co-inhabit with the P-symbiont *Portiera* inside the bacteriocytes and are vertically transmitted via the eggs [30], [31].

The P-symbiont *Portiera* infects every individual of any *B. tabaci* populations. By contrast, the infection frequencies of the six S-symbionts vary greatly among *B. tabaci* populations [31]–[36]. Little, however, is known about the factors affecting the infection dynamics of S-symbionts in *B. tabaci*. In this study, we conducted a systematic survey of symbiotic bacteria in 61 field populations of *B. tabaci* collected from various crops and locations in China. The results obtained suggest that biotype, host plant species, and geographical location of *B. tabaci* are the major factors affecting the population dynamics of S-symbionts in *B. tabaci*.

## Materials and Methods

### *B. tabaci* field populations

**Figure 1:**
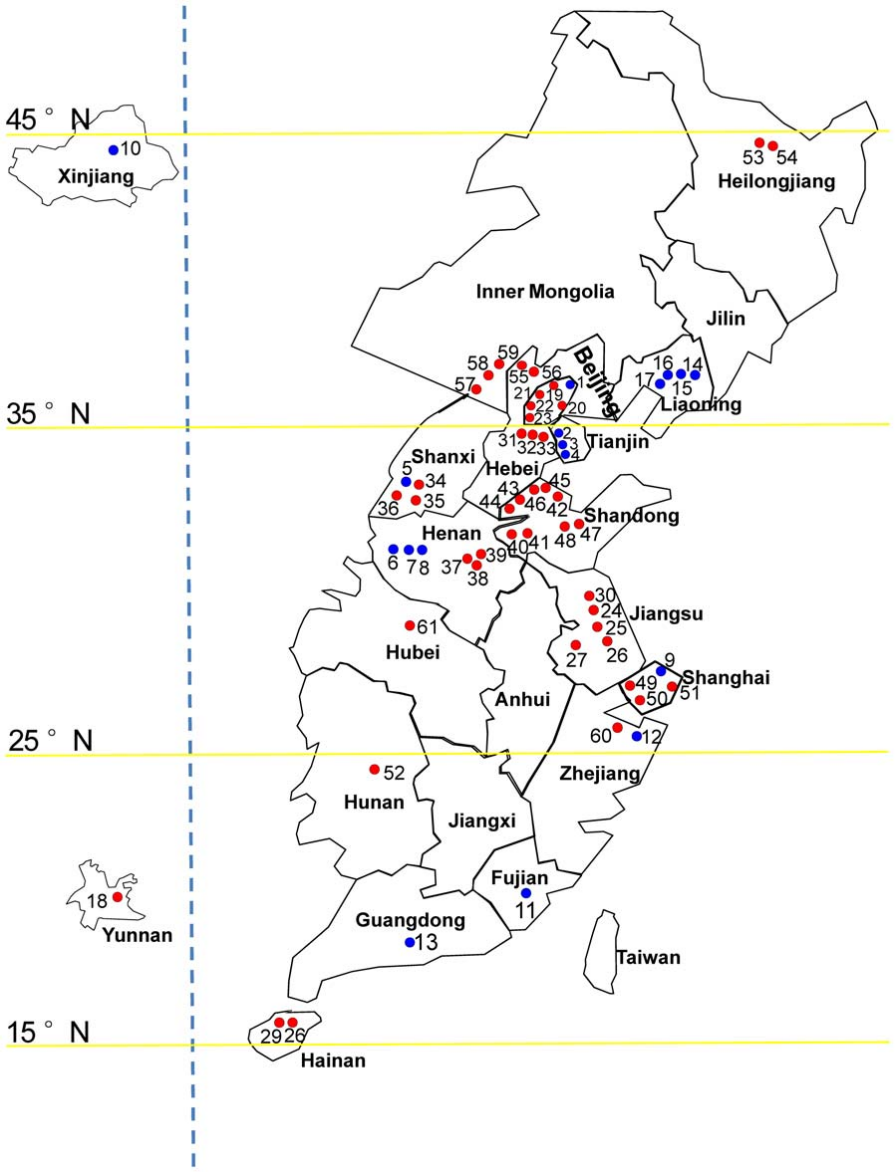
The collection sites of the 61 field populations of *B. tabaci* in China in 2009. The numbers on the map correspond to the codes of the 61 populations in Table S1. The provinces of China in which no samples were collected are not included in this map. The vertical blue dot line indicates deletion of the central provinces between the western and the eastern provinces of China. Beijing, Tianjin and Shanghai cities are disproportionally enlarged. The filled blue and red circles represent B- and Q-biotype populations, respectively.

The 61 *B. tabaci* field populations used in this study were collected from 61 localities in 19 provinces of China in 2009 (Fig. 1). The 61 locations cover an area stretching from Hainan province (16°86′N–110°47′E) in the South to Heilongjiang province (44°83′N–132°53′E) in the North, and from Xinjiang Uygur autonomous region (40°78′N–78°58′E) in the West to Shanghai city (28°18′N–126°46′E) in the East. From each location, dozens of adults were collected alive, fixed in 95% ethanol, and stored at −20°C until analysis of symbionts. The locations, host plants, and biotypes of the 61 field populations are presented in Table S1.

### Biotype determination

Genomic DNA was extracted from each of approximately 24 individual whiteflies (or 24 pairs) per population using the methods of Luo et al. (2002) [21]. Briefly, individual adult was homogenized in 20 µL of extraction buffer (50 mM Tris-HCl, pH 8.0, 20 mM NaCl, 1 mM EDTA, and 1% SDS) on a piece of parafilm. The extract was transferred to a microcentrifuge tube containing 2 µL of proteinase K and incubated at 60°C for 3 h. 78 µL of distilled water was added to the tube, which was incubated at 100°C for 5 min. After incubation, the extract (about 100 µL in total) was mixed well with 200 µL of absolute alcohol. The sample was then placed at −20°C for 2 h and centrifuged at 12,000 rpm for 20 min. The DNA pellets obtained were washed once with 75% ethanol, air dried, dissolved in 20 µL of TE buffer (pH 8.0), and stored at −20°C for subsequent biotyping and PCR detection of symbionts. The biotype of every individual of each population was determined by PCR amplification of *mitochondrial cytochrome oxidase I* (*mtCOI*) gene fragment using biotype-specific primers [37]. The accuracy of such PCR-based biotyping was further verified by sequencing a ∼620-bp *mtCOI* fragment of two individuals per population amplified using the primers C1-J-2195 and TL2-N-2819 [38].

### Screening for the presence of symbionts and data analysis

Approximately 24 (or 24 pairs of) adults each of the 61 field populations (Fig. 1; Table S1) were analyzed for the presense of the P-symbiont *Portiera* and the S-symbionts *Hamiltonella*, *Arsenophonus*, *Cardinium*, *Wolbachia*, *Rickettsia*, and *Fritschea* (Table S1). The presence of *Portiera, Hamiltonella*, *Arsenophonus*, *Cardinium*, *Wolbachia*, *Rickettsia*, and *Fritschea* in each individual adult was determined by PCR-gel analyses of 16S rRNA (*Portiera, Hamiltonella*, *Cardinium*, and *Rickettsia)*, 23S rRNA (*Arsenophonus* and *Fritschea)* or wsp (*Wolbachia)* gene using genus-specific primers (Table 1). PCR amplifications were performed in 25 µL reactions containing 2.5 µL 10×PCR Buffer (Mg^2+^ Plus), 2 µL dNTP mix (2.5 mM of each nucleotide), 0.5 µL of each primer (10 µM each), and 0.125 µL of TaKaRa Taq (5 U/ µL) (TaKaRa Biotechnology (Dalian) Co., Ltd). The PCR cycling conditions for detection of the seven symbionts were summarized in Table 1. The resultant PCR products were electrophoresed with the negative and positive controls of each symbiont on a 1.0% agarose gel in a 0.5×TBE buffer and visualized by ethidium bromide staining. The identities of the expected bands for each of the seven symbionts were verified by subsequent sequencing of two independent clones derived from two different whitefly individuals. As a result, whether an individual adult was infected by any of the seven symbionts was determined based on the presence of the corresponding band.

**Table 1:** PCR primers and conditions used.

Symbiont name	Gene amplified	Primer name	Sequence (5′-3′)	Annealing temperature	Product size (kb)	References
*Portiera*	16S Rdna	28F	TGCAAGTCGAGCGGCATCAT	58°C	∼1	[25]
		1098R	AAAGTTCCCGCCTTATGC GT			
*Rickettsia*	16S rDNA	Rb-F	GCTCAGAACGAACGCTATC	60°C	∼0.9	[28]
		Rb-R	GAAGGAAAGCATCTCTGC			
*Hamiltonella*	16S rDNA	Ham-F	TGAGTAAAGTCTGGAATCTGG	58°C	∼0.7	[25]
		Ham-R	AGTTCAAGACCGCAACCTC			
*Cardinium*	16S rDNA	CFB-F	GCGGTGTAAAATGAGCGTG	57°C	∼0.4	[27]
		CFB-R	ACCTMTTCTTAACTCAAGCCT			
*Wolbachia*	*Wsp*	wsp-81F	TGGTCCAATAAGTGATGAAGAAAC	55°C	∼0.6	[51]
		wsp-691R	AAAAATTAAACGCTACTCCA			
*Fritschea*	23S rDNA	U23F	GATGCCTTGGCATTGATAGGCGATGAAGGA	60°C	∼0.6	[29]
		23SIGR	TGGCTCATCATGCAAAAGGCA			
*Arsenophonus*	23S rDNA	Ars23S-1	CGTTTGATGAATTCATAGTCAAA	58°C	∼0.6	[26]
		Ars23S-2	GGTCCTCCAGTTAGTGTTACCCAAC			

The total numbers of tested and infected adults by the seven symbionts for each field population were recorded to calculate their infection frequencies by the formula: the number of individuals infected or co-infected by a given symbiont or a symbiont combination/the total number of individuals screened (usually n = 24) ×100%. The impacts of biotype, sex, host plant species, and geographical location on the infection frequencies of the symbionts were analyzed using the χ2 tests, followed by multiple comparisons with Bonferroni corrections. The differences between the observed and expected ( =  multiplication of infection frequencies of each symbionts) frequencies of multiple infections were compared by nonparametric χ2 tests. All statistical analyses were performed with SPSS version 18.0 (SPSS Inc., Chicago, IL, USA).

## Results

### 1. Nation-wide survey of symbionts in the field populations of *B. tabaci*

A total of 61 field populations of *B. tabaci* collected from a wide range of host plants in 19 provinces of China during July-September 2009 were biotyped and tested for the diversity and infection frequencies of the P- and S-symbionts (Fig. 1 and Table S1). 44 out of the 61 populations belonged to the Q1 subclade of the Q biotype and the remaining 17 were of pure B biotype (Table S1). All individuals of the 61 field populations had the P-symbiont *Portiera*, regardless of their collection sites, host plants, biotype and sex of the whitefly (Table S1).

Among the 6 S-symbiont species we examined, *Arsenophonus* was not detected in any of the 61 field populations (data not shown). PCR-gel analysis revealed that 24.6% of the 61 populations had the expected band for *Fritschea* (data not shown), but subsequent cloning and sequencing of the band eluted from gels confirmed that it was either *Flavobacterium* or *Acidobacteria*, not *Fritschea* (data not shown). *Wolbachia* was extremely rare and only detected in one B biotype (Code 6 in Table S1) and one Q biotype (Code 52 in Table S1) populations. Interestingly, both populations were collected from cotton and had the same infection frequency (4.2%) by *Wolbachia* (Table S1), but were geographically far from each other (Fig. 1).

*Rickettsia*, *Hamiltonella*, and *Cardinium* were present alone or together in more than 2 field populations (Table S1). The infection frequencies of the three S-symbionts varied greatly among the 61 populations. Statistical analyses revealed that the plant species, geographical location, biotype and sex of the field populations were the four factors significantly contributing to the great variations in the diversity and infection frequency of the S-symbionts in the *B. tabaci* field populations.

### 2. Impacts of *B. tabaci* biotype on the infection frequency of the S-symbionts

The B- and Q-biotype field populations differed significantly in their infection frequencies by the S-symbionts. All the 17 B biotype populations possessed at least one S-symbiont, whereas 4 (i.e. 9.1%; Codes 30, 37, 49, and 56 in Table S1) out of the 44 Q biotype populations did not harbor any of the 6 S-symbionts. The infection frequency of *Wolbachia* was very low in both biotypes and there was no significant difference between the B- and Q- biotype individuals (Table 2). The two biotypes also had a similar percentage of *Hamiltonella* infection. The infection frequency of *Rickettsia* was significantly higher in the 17 B biotype populations (64.3% of a total of 456 B biotype individuals) than the 44 Q biotype populations (7.2% of a total of 1149 Q biotype individuals) (Table 2). By contrast, the infection frequency of *Cardinium* was significantly greater in the 44 Q biotype populations (16.3%) than in the 17 B biotype populations (10.8%). Co-infection of whiteflies by *Wolbachia* and any other S-symbionts was not found in the two biotypes. The B biotype field populations also had significantly higher co-infection frequencies by *Rickettsia + Hamiltonella (RH)* (40.6%), *Rickettsia + Cardinium (RC)* (8.6%), and *Rickettsia + Hamiltonella + Cardinium (RHC)* (5.5%) than the Q biotype field populations (Table 2). In the B biotype, the observed frequency of *RH* co-infection was significantly higher than its expected frequency, which was calculated by multiplying the infection frequencies of each symbionts. No differences were found between the observed and expected frequencies of *RC, HC* and *RHC* co-infections in the two biotypes (Table 2).

**Table 2:** Impacts of biotype on the infection frequency of S-symbionts in the field populations of *B. tabaci* (B biotype: 17 populations, 456 adults in total; Q biotype: 44 populations, 1149 adults in total)*.

	Infection Frequency (%) By											
Biotype	RHC		RH		RC		HC		R	H	C	W
	exp	obs	exp	obs	exp	obs	exp	obs				
B	3.2a	5.5Aa	30.0b	40.6Aa	6.9a	8.6Aa	5.0a	6.8Aa	64.3A	46.7A	10.8B	0.2A
Q	0.5a	0.8Ba	3.3a	4.2Ba	1.2a	1.0Ba	7.6a	6.7Aa	7.2B	46.5A	16.3A	0.0A

*The frequencies in columns sharing the same upper case letter are not significantly different at P<0.05 (multiple comparisons with Bonferroni corrections). The expected (exp) co-infection frequencies of whiteflies by two or three symbionts (e.g. *RH* or *RHC*) were calculated by multiplying the infection frequencies of whiteflies by each of the two or three symbionts. The expected (exp) and observed (obs) co-infection frequencies that share the same lower case letter are not significantly different at P<0.05 (nonparametric tests χ2).

### 3. Impacts of plant species on the infection frequency of S-symbionts

Chi-square tests showed that host plant species significantly and differentially affected the infection frequencies of the B and Q biotypes *B. tabaci* by the S-symbionts (Tables 3 and 4). In the B biotype populations, *Rickettsia* was the most prevalent S-symbiont. It was detected in the B biotype whiteflies collected from all the 7 plant species (cucumber, tomato, cabbage, cotton, poinsettia, sweet potato, and bean; each representing 1 plant family) surveyed (Table S1 and 3). The infection frequency of *Rickettsia* was the highest in the B biotype populations collected from cotton (100%), followed by those from cucumber (73.3%), poinsettia (70.8%), and cabbage (67.7%), those from bean (41.7%) and sweet potato (33.3%), and those from tomato (12.5%) (Table 3). *Hamiltonella* was less prevalent than *Rickettsia* in the B biotype populations and was not detected in the B biotype whiteflies from sweet potato (Table 3). Chi-square tests divided the B biotype whiteflies from the 6 plants into 2 significantly different groups. The B biotype populations from poinsettia (83.3%), cotton (61.5%), cucumber (57.5%) and cabbage (50.0%) had significantly higher *Hamiltonella* infection than those from tomato (20.8%) and bean (8.3%) (Table 3). *Cardinium* was detected only in two B biotype populations, one from cucumber and another from cotton (Table S1). The infection frequency of *Cardinium* was significantly higher in the cucumber population than in the cotton population (Table 3). As a result, the co-infection rates of *RC*, *HC* and *RHC* were higher (significant for *RHC* and *HC*) in the cucumber population than in the cotton population (Table 3). *RH* co-infection was significantly more prevalent in the cucumber, cabbage, cotton, and poinsettia populations than in the tomato, bean and sweet potato populations. Further nonparametric χ2 tests revealed no significant differences between the observed and expected frequencies of *RHC*, *RC* and *HC* co-infection in the populations from all the seven plant species (Table 3).

**Table 3:** Impacts of host plant on the diversity and infection frequency of S-symbionts in the field populations of B biotype *B. tabaci* *.

	Infection Frequency (%) By											
Host plant	RHC		RH		RC		HC		R	H	C	W
	exp	obs	exp	obs	exp	obs	exp	obs				
Cucumber	14.0a	18.3Aa	42.1a	51.7Aa	24.4a	25.0Aa	19.1a	23.3Aa	73.3B	57.5A	33.3A	0.0A
Tomato	0.0a	0.0Ba	2.6a	6.9Ba	0.0a	0.0Ba	0.0a	0.0Ba	12.5E	20.8B	0.0C	0.0A
Cabbage	0.0a	0.0Ba	33.9a	45.8Aa	0.0a	0.0Ba	0.0a	0.0Ba	67.7BC	50.0A	0.0C	0.0A
Cotton	5.8a	3.1Ba	61.5a	60.4Aa	9.4a	9.4Aa	5.8a	3.1Ba	100.0A	61.5A	9.4B	1.0A
Poinsettia	0.0a	0.0Ba	59.0a	62.5Aa	0.0a	0.0Ba	0.0a	0.0Ba	70.8BC	83.3A	0.0C	0.0A
Sweet potato	0.0a	0.0Ba	0.0a	0.0Ba	0.0a	0.0Ba	0.0a	0.0Ba	33.3DE	0.0B	0.0C	0.0A
Bean	0.0a	0.0Ba	3.5a	4.2Ba	0.0a	0.0Ba	0.0a	0.0Ba	41.7CD	8.3B	0.0C	0.0A

*The frequencies in columns sharing the same upper case letter are not significantly different at P<0.0024 (multiple comparisons with Bonferroni corrections). The expected (exp) and observed (obs) co-infection frequencies that share the same lower case letter are not significantly different at P<0.05 (nonparametric tests χ2).

**Table 4:** Impacts of host plant on the diversity and infection frequency of S-symbionts in the field populations of Q biotype *B. tabaci* *.

	Infection Frequency (%) By											
Host plant	RHC		RH		RC		HC		R	H	C	W
	exp	obs	Exp	obs	exp	obs	exp	obs				
Cucumber	0.0a	0.0Aa	0.4a	0.0Ca	0.3a	0.0Aa	1.8a	1.9Ba	2.3C	16.4CD	11.2BC	0.0A
Towel gourd	2.0a	4.4Aa	9.1a	13.0Ba	5.7a	4.4Aa	7.6a	17.4Aa	26.1AB	34.8BCD	21.7AB	0.0A
Squash	0.0a	0.0Aa	0.0a	0.0Ca	0.0a	0.0Aa	0.0a	0.0Ba	4.2BC	0.0D	0.0D	0.0A
Tomato	0.0a	0.0Aa	0.0a	0.0Ca	0.0a	0.0Aa	5.7a	7.3ABa	0.0C	32.3BCD	17.7BC	0.0A
Pepper	0.0a	0.0Aa	0.0a	0.0Ca	0.0a	0.0Aa	31.8a	19.4Ab	0.0C	61.1AB	52.1A	0.0A
Eggplant	0.7a	1.5Aa	5.4a	6.0Ba	1.3a	2.1Aa	6.9a	6.0ABa	10.1B	53.6AB	12.8BC	0.0A
Cotton	0.0a	0.0Aa	5.7a	5.4BCa	0.1a	0.0Aa	0.3a	0.6Ba	11.3B	50.6AB	0.6D	0.6A
Japanese hop	0.1a	1.4Aa	1.1a	1.4BCa	0.1a	1.4Aa	6.4a	6.9ABa	1.4C	76.4A	8.4BCD	0.0A
Poinsettia	0.0a	0.0Aa	0.0a	0.0Ca	0.0a	0.0Aa	24.3a	0.0Bb	0.0C	83.3A	29.2AB	0.0A
Gerbera	16.4a	8.3Aa	65.6a	62.5Aa	18.8a	12.5Aa	21.9a	20.8Aa	75.0A	87.5A	25.0AB	0.0A
Bean	0.0a	0.0Aa	0.0a	0.0Ca	0.0a	0.0Aa	5.2a	8.3ABa	0.0C	41.7ABC	12.5BCD	0.0A

*The frequencies in columns sharing the same upper case letter are not significantly different at P<0.00091 (multiple comparisons with Bonferroni corrections). The expected (exp) and observed (obs) co-infection frequencies that share the same lower case letter are not significantly different at P<0.05 (nonparametric tests χ2).

Unlike in the B biotype populations, *Rickettsia* was the least prevalent S-symbiont in the Q biotype whiteflies (Table S1 and Table 4). It was found in the Q biotype populations from gerbera, Japanese hop, cotton, egg plant, squash, towel gourd, and cucumber, but not from tomato, pepper, poinsettia, and bean (Table 4). *Rickettsia* infection was the highest in the Q biotype whiteflies from Gerbera (75.0%), followed by those from towel gourd (26.1%), those from cotton (11.3%) and eggplant (10.1%), and those from squash (4.2%), cucumber (2.3%) and Japanese hop (1.4%). *Hamiltonella* and *Cardinium* were more prevalent and detected in the Q biotype populations from all the 11 plants but squash. Q biotype whiteflies from gerbera (87.5%), poinsettia (83.3%) and Japanese hop (76.4%) had the highest rate of *Hamiltonella* infection, followed by those from pepper (61.1%), eggplant (53.6%), cotton (50.6%), and bean (41.7%), those from towel gourd (34.8%) and tomato (32.3%), and those from cucumber (16.4%) and squash (0.0%). For *Cardinium*, the pepper populations (52.1%) had the highest infection frequency, whereas the squash (0.0%) and cotton (0.6%) populations had the lowest infection (Table 4). The plant populations between the two groups had a more or less similar level of *Cardinium* infection. Further, Q whiteflies from gerbera and towel gourd had a greater frequency of *RH* (significant), *RC* and *RHC* co-infection than those from other plants (Table 4). For *HC* co-infection, Q whiteflies from gerbera, pepper and towel gourd had the greatest frequency, followed by those from bean, tomato, Japanese hop, and eggplant, and those from the remaining plants. Nonparametric χ2 tests showed no significant differences between the observed and expected frequencies of *RH*, *RC* and *RHC* co-infections in the populations from all the seven plant species (Table 4). The observed frequencies of *HC* co-infection were significantly lower than its expected frequencies in the pepper and poinsettia populations (Table 4).

### 4. Impacts of geographical location on the infection frequency of S-symbionts

To test the effects of geographical location on the infection and co-infection of the 4 S-symbionts, we divided the collection sites into three geographical regions: 15°N–25°N, 25°N–35°N and 35°N–45°N (Fig. 1). The B biotype populations collected from the three regions had a similar level of *Rickettsia* and *Hamiltonella* infection frequency (Table 5). By contrast, *Cardinium* and *Wolbachia* were found only in the 35°N–45°N region (18.6%, significantly greater than 0.0% in the other two regions) and the 25°N–35°N region (0.7%, not significantly different from 0.0% in the two other regions), respectively. Consequently, *RC*, *HC*, and *RHC* co-infections of the B biotype whiteflies existed only in the 35°N–45°N region; the corresponding co-infection frequency was significantly greater than 0.0% in the two southern regions. In contrast, *RH* co-infection was prevalent in all the regions, but no significant difference in co-infection frequency existed between the three regions (Table 5). In addition, the observed co-infection frequencies of *HC* and *RHC* in the 35°N–45°N region and *RH* in the 25°N–35°N and 35°N–45°N regions were significantly greater than their expected frequencies.

**Table 5:** Impacts of geographical location on the diversity and infection frequency of S-symbionts in the field populations of B biotype *B. tabaci* *.

	Infection Frequency (%) By												
Location	RHC		RH		RC		HC		R	H	C	W	
	exp	obs	Exp	obs	exp	obs	exp	obs					
15°N–25°N	0.0a	0.0Ba	21.7a	31.3Aa	0.0a	0.0Ba	0.0a	0.0Ba	52.1A	41.7A	0.0B	0.0A	
25°N–35°N	0.0a	0.0Ba	36.4b	46.5Aa	0.0a	0.0Ba	0.0a	0.0Ba	64.6A	56.3A	0.0B	0.7A	
35°N–45°N	5.2b	9.5Aa	28.1b	34.9Aa	12.3a	14.8Aa	7.9b	11.7Aa	66.3A	42.4A	18.6A	0.0A	

*The frequencies in columns sharing the same upper case letter are not significantly different at P<0.017 (multiple comparisons with Bonferroni corrections). The expected (exp) and observed (obs) co-infection frequencies that share the same lower case letter are not significantly different at P<0.05 (nonparametric tests χ2).

In the Q biotype whiteflies, *Rickettsia*, *Hamiltonella* and *Cardinium* were present in the three geographical regions, whereas *Wolbachia* (1.1%) was detected only in the 15°N–25°N region (Table 6). *Hamiltonella* was prevalent in the three geographical regions, with no significant difference in the infection rate among the three regions (χ2 = 2.821, *P* = 0.244). The infection rate of *Rickettsia* increased significantly from the North to the South. The infection rate of *Cardinium* was the highest in the 35°N–45°N region, followed by the 15°N–25°N region, and the 25°N–35°N region (Table 6). Double or triple co-infection rate of Q biotype whiteflies by the three S-symbionts ranged from 0.4% to 5.9% across the three regions, with no significant difference. There were no significant differences between the observed and expected frequencies of *RH, RC* and *RHC* in all the three regions. But the observed frequency of *HC* in the 35°N–45°N region was significantly lower than its expected frequency (Table 6).

**Table 6:** Impacts of geographical location on the diversity and infection frequency of S-symbionts in the field populations of Q biotype *B. tabaci* *.

	Infection Frequency (%) By											
Location	RHC		RH		RC		HC		R	H	C	W
	exp	obs	exp	obs	exp	obs	exp	obs				
15°N–25°N	1.1a	1.1Aa	8.4a	4.2Aa	2.5a	1.1Aa	5.3a	4.2Aa	20.0A	42.1A	12.6B	1.1A
25°N–35°N	0.2a	0.4Aa	3.9a	4.5Aa	0.3a	0.6Aa	2.2a	2.4Aa	7.5B	52.2A	4.3C	0.0B
35°N–45°N	0.3a	1.4Aa	0.7a	2.0Aa	0.7a	1.4Aa	12.6a	5.9Ab	2.0C	36.6A	34.4A	0.0B

*The frequencies in columns sharing the same upper case letter are not significantly different at P<0.017 (multiple comparisons with Bonferroni corrections). The expected (exp) and observed (obs) co-infection frequencies that share the same lower case letter are not significantly different at P<0.05 (nonparametric tests χ2).

### 5. Impacts of sex on the infection frequency of S-symbionts

Male and female adults of 2 B- (Codes 1 and 2 in Table S1) and 4 Q-biotype (Codes 19, 20, 22, and 23 in Table S1) field populations were separately screened for the presence of S-symbionts. χ2 tests indicated that sex significantly affected the infection frequency of *Hamiltonella*, but not *Rickettsia* or *Cardinium* (Table 7). The average infection frequency of *Hamiltonella* was significantly higher in females (88.9%) than in males (12.5%) (χ2 = 165.046, *P*<0.0001). The co-infection frequencies of *RHC*, *RH*, and *HC*, but not *RC*, were also significantly higher in females than in males (*P*<0.0001). No significant differences existed between the observed and expected co-infection frequencies of female and male whiteflies by *RH*, *RC*, and *RHC*. The observed frequency of *HC* was significantly lower than its expected frequency in female, but not male whiteflies (Table 7).

**Table 7:** Impacts of sex on the diversity and infection frequency of S-symbionts in the 6 field populations (population codes 1, 2, 19, 20, 22, and 23 in Table S1) of B. tabaci*.

	Infection Frequency (%) By										
Sex	RHC		RH		RC		HC		R	H	C
	Exp	obs	exp	obs	exp	obs	exp	obs			
♀	17.8a	17.4Aa	30.2a	33.3Aa	20.1a	17.4Aa	52.5a	29.2Ab	34.0A	88.9A	59.0A
*♂*	2.4a	3.5Ba	4.1a	6.3Ba	19.5a	14.6Aa	7.5a	3.5Ba	32.6A	12.5B	59.7A

• The frequencies in columns sharing the same upper case letter are not significantly different at P<0.05 (multiple comparisons with Bonferroni corrections). The expected (exp) and observed (obs) co-infection frequencies that share the same lower case letter are not significantly different at P<0.05 (nonparametric tests χ2).

## Discussion

Several recent studies have showed a 100% infection frequency of *B. tabaci* by the P-symbionat *Portiera*, but a 0–100% infection frequency by any of the six S-symbionts (*Hamiltonella*, *Arsenophonus*, *Cardinium*, *Wolbachia*, *Rickettsia* and *Fritschea)*, depending on the populations tested [31]–[36]. To reveal the key factors that govern the infection dynamics of whiteflies by the six S-symbionts, we surveyed 61 field populations of (24 individuals or 24 pairs each) for the presence of the P-symbiont *Portiera* and the six S-symbionts. Consistent with the aforementioned studies, all individuals of the 61 field populations harbored the P-symbiont *Portiera. Arsenophonus* and *Fritschea* were not present in any of the 61 populations, whereas the presence and infection frequencies of *Hamiltonella*, *Cardinium*, *Wolbachia* and *Rickettsia* varied greatly among the 61 populations.

Our data from the 61 (17 B biotype and 44 Q biotype) field populations and all the prior studies [31]–[36] demonstrate that biotype or genetic group of *B. tabaci* is a key factor determining the infection dynamics of the six S-symbionts. However, there are noticeable discrepancies in the S-symbiont-host biotype associations or linkage disequilibrium revealed by these studies. For example, Chiel et al. (2007) and Gueguen et al. (2010) reported that the B biotype only harbored *Hamiltonella* (100%) and *Rickettsia* (64.0%), the Q1 subclade of the Q biotype only harbored *Hamiltonella* (close to 100.0%), *Cardinium* and *Wolbachia*, the Q2 subclade only harbored *Rickettsia* (74.0%), *Wolbachia* (33.0%) and *Arsenophonus* (87.0%), and the Q3 subclade only harbored *Rickettsia* (about 30.0%) and *Arsenophonus* (about 97%). By contrast, all of the six S-symbionts but *Fritschea* were detected in biotypes B and Q (i.e. Q1) in China during 2005 to 2009 [36], and the Q biotype in Croatia [31] hosted *Hamiltonella*, *Rickettsia*, *Wolbachia* and *Cardinium*, but not *Arsenophonus* and *Fritschea*.

One explanation for the above discrepancies is that there are other important factors that have not been characterized but significantly affect the infection dynamics of the S-symbionts in *B. tabaci*. That the female adults of 2 B- (Codes 1 and 2 in Table S1) and 4 Q-biotype (Codes 19, 20, 22, and 23 in Table S1) field populations had significantly higher infection frequencies of *Hamiltonella, RHC*, *RH*, and *HC* than did their male adults (Table 7) suggests that sex of the hosts could be another factor impacting the infection dynamics of *B. tabaci* by the 6 S-symbionts. However, this female-biased infection rate is probably not a real infection difference between male and female adults, but a matter of a combination of lower symbiont titer in males and lower detection sensitivity of our PCR-gel analysis technique. Single whiteflies are small and ethidium bromide used in this study is not the most sensitive DNA stain. It is well known that symbiont infections are not easily detectable in arthropods [39]. Moreover, it is females, not males, that transmit the symbionts to their offspring, and males are smaller than females. Therefore, it makes some sense that the titer of symbionts in males would be lower and thus would be more difficult to be detected by our PCR-gel analysis technique. Another less likely explanation for the pattern of lower frequencies of symbiont infections in males would be male-killing effects of *Hamiltonella*, *RHC*, *RH*, and *HC*. The fact that the male-killing symbiont identified in the ladybird *Cheilomenes sexmaculata* shares the closest similarity to *Hamiltonella defensa* (98% nucleotide identity) of whiteflies [40] implicates that *Hamiltonella* of whiteflies could be a male-killing symbiont. More experiments are necessary to resolve these two possibilities.

Our survey data of the 61 field population collected from three different regions in China (15°N–25°N, 25°N–35°N, and 35°N–45°N) suggest that geographical location is another factor affecting the infection frequency of the S-symbionts in *B. tabaci*. The impacts of geographical locations are best exemplified by infection of the B biotype by *Cardinium* and *Wolbachia* only in the 35°N–45°N and the 25°N–35°N regions respectively (Table 5), significantly greater infection of the Q biotype by *Cardinium* in the 35°N–45°N region, and the increase of *Rickettsia* infection with decreasing latitude in the Q biotype (Table 6). This is consistent with the results of Ahmad et al. (2010), who showed that the infection frequency of *Wolbachia* in *B. tabaci* was related to country (location).

Our survey data of the 61 field populations collected from 7 (B biotype) or 11 (Q biotype) different plant species also indicate that host plant affects the population dynamics of the S-symbionts in *B. tabaci*. This is because the infection frequencies of *Rickettsia, Cardinium*, *RH*, and *HC* were all significantly different among the B and Q biotype field populations collected from different plant species (Table 3 and 4). This agrees with the findings of Chiel et al. (2007), who reported that *Rickettsia* and *Arsenophonus* had a significantly higher infection frequency in the Q populations collected from sage than those from all other host plants.

In summary, at least three factors including biotype or genetic group, host plant and geographical location affect the infection dynamics of S-symbionts in *B. tabaci*. Two of the three factors—host plants [6], [41]–[45] and collection sites [42], [46]—as well as temperature [46]–[48] are known to affect the infection frequency of S-symbionts in other insects. Nonetheless, the host plant- and/or location-symbiont association patterns revealed from the field populations could be caused, at least partially, by factors other than host plants or latitude, particularly population inertia and stochasticity. In theory, these genetic (biotype, sex) and environmental factors (host plant and geographical location) could change the horizontal transfer frequency, vertical transmission fidelity, and/or relative fitness of these S-symbionts on *B. tabaci*. Further experiments are required to address how the four factors affect the population dynamics of the six maternally transmitted S-symbionts in *B. tabaci*.

This study showed that co-infections with *Rickettsia* and *Hamiltonella* are more common than expected in natural populations of B biotype *B. tabaci* (Table 2–3, 5). In theory, there are a number of possible mechanisms that can facilitate such endosymbiont co-infections. For example, the co-infecting endosymbionts may additively confer fitness advantages on the host, either by positively affecting different fitness parameters or by favoring overlapping fitness parameters in a synergistic way [49]. However, the co-infections by *HC* are significantly rarer than expected in natural populations of B- and Q-biotype *B. tabaci* (Table 4, 6–7), suggesting antagonistic interactions between the S-symbionts upon co-infection, possibly because of competition for limited resource and niche in the same host individual [30], [50] or synergistic negative effects of co-infection on the host organism [8]. To confirm which of these hypotheses can better account for the co-infection patterns for each of the S-symbiont pairs, further experiments are needed.

## Supporting Information

Table S1 The locations, host plants, biotypes, and symbionts of the 61 field populations of B. tabaci collected in 2009^*^.
(DOC)
