# Supplementary material for: Factors Affecting Population Dynamics of Maternally Transmitted Endosymbionts in Bemisia tabaci
Source: PLoS One. 2012 Feb 23;7(2):e30760. doi: 10.1371/journal.pone.0030760 (PMC3285672; doi:10.1371/journal.pone.0030760)
Supplement: Table S1 — The locations, host plants, biotypes, and symbionts of the 61 field populations of B. tabaci collected in 2009*. (DOC) [file pone.0030760.s001.doc]

**Table S1** The locations, host plants, biotypes, and symbionts of the 61 field populations of *B. tabaci* collected in 2009*.

|  |  |  |  |  |  | Infection Frequency (%) By | | | | | |  |  |
| --- | --- | --- | --- | --- | --- | --- | --- | --- | --- | --- | --- | --- | --- |
| Population code | Location | Host plant | Biotype | Sex | N | RHC | RH | RC | HC | R | H | C | W |
| 1 | Haidian, Beijing | Cucumber | B | ♀ | 24 | 75.0 | 75.0 | 75.0 | 100.0 | 75.0 | 100.0 | 100.0 | - |
|  |  |  | B | *♂* | 24 | 16.7 | 25.0 | 50.0 | 16.7 | 75.0 | 29.2 | 66.7 | - |
| 2 | Xiqing, Tianjin | Cotton | B | ♀ | 24 | 8.3 | 95.8 | 8.3 | 8.3 | 100.0 | 95.8 | 8.3 | - |
|  |  |  | B | *♂* | 24 | 4.2 | 12.5 | 29.2 | 4.2 | 100.0 | 12.5 | 29.2 |  |
| 3 | Xiqing, Tianjin | Cucumber | B | Mix | 24 | - | 66.7 | - | - | 100.0 | 66.7 | - | - |
| 4 | Xiqing, Tianjin | Cabbage | B | Mix | 24 | - | 100.0 | - | - | 100.0 | 100.0 | - | - |
| 5 | Yucheng, Shanxi | Tomato | B | ♀ | 24 | - | - | - | - | - | 12.5 | - | - |
| 6 | Luoyang, Henan | Cotton | B | Mix | 24 | - | 83.3 | - | - | 100.0 | 83.3 | - | 4.2 |
| 7 | Luoyang, Henan | Cucumber | B | Mix | 24 | - | 91.7 | - | - | 100.0 | 91.7 | - | - |
| 8 | Luoyang, Henan | Cabbage | B | Mix | 24 | - | 79.2 | - | - | 87.5 | 91.7 | - | - |
| 9 | Shanghai | Cabbage | B | Mix | 24 | - | 4.2 | - | - | 75.0 | 8.3 | - | - |
| 10 | Urumchi, Xinjiang | Cotton | B | Mix | 24 | - | 50.0 | - | - | 100.0 | 54.2 | - | - |
| 11 | Fuzhou, Fujian | Sweet potato | B | Mix | 24 | - | - | - | - | 33.3 | - | - | - |
| 12 | Hangzhou, Zhejiang | Tomato | B | Mix | 24 | - | 20.8 | - | - | 25.0 | 50.0 | - | - |
| 13 | Guangzhou, Guangdong | Poinsettia | B | Mix | 24 | - | 62.5 | - | - | 70.8 | 83.3 | - | - |
| 14 | Shenyang, Liaoning | Cucumber | B | Mix | 24 | - | - | - | - | 16.7 | - | - | - |
| 15 | Shenyang, Liaoning | Tomato | B | Mix | 24 | - | - | - | - | 12.5 | - | - | - |
| 16 | Shenyang, Liaoning | Beans | B | Mix | 24 | - | 4.2 | - | - | 41.7 | 8.3 | - | - |
| 17 | Shenyang, Liaoning | Cabbage | B | Mix | 24 | - | - | - | - | 8.3 | - | - | - |
| 18 | Kunixming, Yunnan | Poinsettia | Q | Mix | 24 | - | - | - | - | - | 83.8 | 29.2 | - |
| 19 | Haidian, Beijing | Pepper | Q | ♀ | 24 | - | - | - | 66.7 | - | 100.0 | 66.7 | - |
|  |  |  | Q | *♂* | 24 | - | - | - | - | - | 16.7 | 66.7 | - |
| 20 | Haidian, Beijing | Pepper | Q | ♀ | 24 | - | - | - | 20.8 | - | 50.0 | 66.7 | - |
|  |  |  | Q | *♂* | 24 | - | - | - | - | - | 16.7 | 83.3 | - |
| 21 | Changping, Beijing | Cucumber | Q | ♀ | 22 | - | - | - | - | - | 13.6 | - | - |
| 22 | Haidian, Beijing | Tomato | Q | ♀ | 24 | - | - | - | 25.0 | - | 100.0 | 25.0 | - |
|  |  |  | Q | *♂* | 24 | - | - | - | - | - | - | 33.3 | - |
| 23 | Haidian, Beijing | Eggplant | Q | ♀ | 24 | 20.8 | 29.2 | 20.8 | 66.7 | 29.2 | 87.5 | 83.3 | - |
|  |  |  | Q | *♂* | 24 | - | - | 8.3 | - | 20.8 | - | 79.2 | - |
| 24 | Yangzhou, Jiangsu | Pepper | Q | ♀ | 24 | - | - | - | 8.3 | - | 83.3 | 8.3 | - |
| 25 | Yangzhou, Jiangsu | Eggplant | Q | ♀ | 24 | - | - | - | - | - | 79.2 | - | - |
| 26 | Yangzhou, Jiangsu | Gerbera | Q | ♀ | 24 | 8.3 | 62.5 | 12.5 | 20.8 | 75.0 | 87.5 | 25.0 | - |
| 27 | Wuxi, Jiangsu | Cucumber | Q | ♀ | 24 | - | - | - | - | - | - | 12.5 | - |
| 28 | Haikou, Hainan | Eggplant | Q | ♀ | 24 | - | 4.2 | - | - | 12.5 | 50.0 | - | - |
| 29 | Haikou, Hainan | Towel Gourd | Q | Mix | 23 | 4.4 | 13.0 | 4.4 | 17.4 | 26.1 | 34.8 | 21.7 | - |
| 30 | Dongtai, Jiangsu | Eggplant | Q | ♀ | 24 | - | - | - | - | - | - | - | - |
| 31 | Langfang, Hebei | Pepper | Q | ♀ | 24 | - | - | - | 20.8 | - | 100.0 | 20.8 | - |
| 32 | Langfang, Hebei | Eggplant | Q | ♀ | 24 | - | - | - | 8.3 | - | 100.0 | 8.3 | - |
| 33 | Langfang, Hebei | Cucumber | Q | ♀ | 24 | - | - | - | - | - | 45.8 | 12.5 | - |
| 34 | Yucheng, Shanxi | Cucumber | Q | ♀ | 24 | - | - | - | - | 8.3 | - | - | - |
| 35 | Yucheng, Shanxi | Eggplant | Q | ♀ | 24 | - | 29.2 | - | - | 41.7 | 66.7 | - | - |
| 36 | Yucheng, Shanxi | Cotton | Q | ♀ | 24 | - | 8.3 | - | - | 20.8 | 45.8 | - | - |
| 37 | Zhengzhou, Henan | Cucumber | Q | Mix | 24 | - | - | - | - | - | - | - | - |
| 38 | Zhengzhou, Henan | Cotton | Q | Mix | 24 | - | - | - | - | 4.2 | - | - | - |
| 39 | Zhengzhou, Henan | Eggplant | Q | Mix | 24 | - | - | - | - | - | 8.3 | - | - |
| 40 | Heze, Shandong | Cotton | Q | Mix | 24 | - | 25.0 | - | - | 4.2 | 83.3 | - | - |
| 41 | Heze, Shandong | Eggplant | Q | Mix | 24 | - | 20.8 | - | - | 20.8 | 100.0 | - |  |
| 42 | Dezhou, Shandong | Eggplant | Q | Mix | 24 | - | - | - | - | - | 83.3 | - | - |
| 43 | Liaocheng, Shandong | Eggplant | Q | Mix | 24 | - | - | - | 8.3 | - | 100.0 | 8.3 | - |
| 44 | Liaocheng, Shandong | Japanese hop | Q | Mix | 24 | 4.2 | 4.2 | 4.2 | 16.7 | 4.2 | 79.2 | 16.7 | - |
| 45 | Dezhou, Shandong | Cotton | Q | Mix | 24 | - | - | - | 4.2 | - | 82.6 | 4.2 | - |
| 46 | Dezhou, Shandong | Japanese hop | Q | Mix | 24 | - | - | - | 4.2 | - | 100.0 | 4.2 | - |
| 47 | Jinan, Shandong | Japanese hop | Q | Mix | 24 | - | - | - | - | - | 50.0 | 4.2 | - |
| 48 | Jinan, Shandong | Cotton | Q | Mix | 24 | - | - | - | - | - | 91.3 | - | - |
| 49 | Shanghai | Tomato | Q | Mix | 24 | - | - | - | - | - | - | - | - |
| 50 | Shanghai | Eggplant | Q | Mix | 24 | - | - | - | - | 16.7 | - | - | - |
| 51 | Shanghai | Cucumber | Q | Mix | 24 | - | - | - | - | 12.5 | - | - | - |
| 52 | Changsha, Hunan | Cotton | Q | Mix | 24 | - | - | - | - | 41.7 | - | - | 4.2 |
| 53 | Haerbin, Heilongjiang | Cucumber | Q | Mix | 24 | - | - | - | 16.7 | - | 20.8 | 62.5 | - |
| 54 | Haerbin, Heilongjiang | Tomato | Q | Mix | 24 | - | - | - | 4.2 | - | 29.2 | 12.5 | - |
| 55 | Zhangjiakou, Hebei | Cucumber | Q | Mix | 24 | - | - | - | - | - | 20.8 | 8.3 | - |
| 56 | Zhangjiakou, Hebei | Eggplant | Q | Mix | 24 | - | - | - | - | - | - | - | - |
| 57 | Huhehaote,  Inner Mongolia | Cucumber | Q | Mix | 24 | - | - | - | - | - | 45.8 | 4.2 | - |
| 58 | Huhehaote,  Inner Mongolia | Squash | Q | Mix | 24 | - | - | - | - | - | 4.2 | - | - |
| 59 | Huhehaote,  Inner Mongolia | Beans | Q | Mix | 24 | - | - | - | 8.3 | - | 41.7 | 12.5 | - |
| 60 | Hangzhou , Zhejiang | Eggplant | Q | Mix | 24 | - | - | - | - | - | 75.0 | - | - |
| 61 | Wuhan , Hubei | Cotton | Q | Mix | 24 | - | 4.2 | - | - | 8.3 | 50.0 | - | - |

* Mix = female and male adults of the samples were not sexed; P = *Portiera*; R = *Rickettsia*; H = *Hamiltonella*; C = *Cardinium*; W = *Wolbachia.*
